# Archetypes of Gamification: Analysis of mHealth Apps

**DOI:** 10.2196/19280

**Published:** 2020-10-19

**Authors:** Manuel Schmidt-Kraepelin, Philipp A Toussaint, Scott Thiebes, Juho Hamari, Ali Sunyaev

**Affiliations:** 1 Department of Economics and Management Karlsruhe Institute of Technology Karlsruhe Germany; 2 Gamification Group Faculty of Information Technology and Communication Sciences Tampere University Tampere Finland

**Keywords:** mHealth, smartphones, mobile phones, gamification, quantified-self, exergames, persuasive technology

## Abstract

**Background:**

Nowadays, numerous health-related mobile apps implement gamification in an attempt to draw on the motivational potential of video games and thereby increase user engagement or foster certain health behaviors. However, research on effective gamification is still in its infancy and researchers increasingly recognize methodological shortcomings of existing studies. What we actually know about the phenomenon today stems from fragmented pieces of knowledge, and a variety of different perspectives. Existing research primarily draws on conceptual knowledge that is gained from research prototypes, and isolated from industry best practices. We still lack knowledge on how gamification has been successfully designed and implemented within the industry and whether certain gamification approaches have shown to be particularly suitable for certain health behaviors.

**Objective:**

We address this lack of knowledge concerning best practices in the design and implementation of gamification for health-related mobile apps by identifying archetypes of gamification approaches that have emerged in pertinent health-related mobile apps and analyzing to what extent those gamification approaches are influenced by the underlying desired health-related outcomes.

**Methods:**

A 3-step research approach is employed. As a first step, a database of 143 pertinent gamified health-related mobile apps from the Apple App Store and Google Play Store is set up. Second, the gamification approach of each app within the database is classified based on an established taxonomy for gamification in health-related apps. Finally, a 2-step cluster analysis is conducted in order to identify archetypes of the most dominant gamification approaches in pertinent gamified health-related mobile apps.

**Results:**

Eight archetypes of gamification emerged from the analysis of health-related mobile apps: (1) competition and collaboration, (2) pursuing self-set goals without rewards, (3) episodical compliance tracking, (4) inherent gamification for external goals, (5) internal rewards for self-set goals, (6) continuous assistance through positive reinforcement, (7) positive and negative reinforcement without rewards, and (8) progressive gamification for health professionals. The results indicate a close relationship between the identified archetypes and the actual health behavior that is being targeted.

**Conclusions:**

By unveiling salient best practices and discussing their relationship to targeted health behaviors, this study contributes to a more profound understanding of gamification in mobile health. The results can serve as a foundation for future research that advances the knowledge on how gamification may positively influence health behavior change and guide practitioners in the design and development of highly motivating and effective health-related mobile health apps.

## Introduction

Following the proliferation of smartphones and other smart devices into people’s everyday lives during the last decade, mobile app stores (eg, the Apple App Store and Google Play Store) now provide users with a plethora of different health-related mobile apps (mHealth apps) [1]. Typical mHealth apps available on these stores include apps that support users in pursuing healthy diets [2], apps that motivate their users to increase their physical activity [3], or apps that help managing chronic diseases properly [4], to name but a few. Surveys show that 58.23% (934/1604) of US smartphone users have downloaded at least one such mHealth app to their smartphones in the past [5,6], making mHealth apps a multibillion Dollar business with annual growth rates of 30% or more [7]. Yet, despite mHealth apps’ potential to positively influence users’ health behavior and their high download numbers, research also suggests that in the past a majority of users have failed to frequently use such apps or even stopped using them after a short period, for example, due to high data entry burden, or loss of interest [5,8].

Gamification presents itself as a promising approach to overcome a loss of interest, increase user engagement [9], raise the quality of health behaviors [10], and motivate users to use mHealth apps for a sustained period [11]. It refers to the overall proliferation of games in culture, society, and technology and describes that technologies are being transformed and designed to afford positive experiences, motivational enforcement, and skill accruement [12]. Drawing on the motivational potential of (video) games to foster certain health behavior outcomes has a long tradition in health care. The development of so-called serious games (ie, “games that do not have entertainment, enjoyment or fun as their primary purpose” [13]), for example, can be traced back as far as to the 1970s [14]. Standing in the long tradition of gaming in health care, gamification has rapidly gained interest by health care researchers and professionals over the last decade. While some health behaviors, such as exercise and exercise programs in themselves are pervasively gameful in nature, mHealth apps supporting such behaviors are increasingly and more explicitly gamified further [15].

Research on gamification and its design is still in its infancy and rapidly evolving. Researchers increasingly recognize methodological shortcomings of existing studies on the effectiveness of gamification and that research on gamification has mainly advanced without an agenda, theoretical guidance, or a clear picture of the field [16,17]. What we actually know about the phenomenon today stems from fragmented pieces of knowledge, and a variety of different perspectives [16]. One of these perspectives has led to the development of a variety of frameworks and processes for designing and implementing effective gamification (see Mora et al [18] and Morschheuser et al [19] for an overview). Another perspective is concerned with providing means (eg, taxonomies) to conceptually classify existing gamification approaches (eg, [20-22]). However, these research streams usually produce research results based on research prototypes. While experiments on simple prototypes of gamification may improve the internal validity of the corpus of gamification research on how any specific gamification features affect behavior, the state of the apps of gamification in practice may differ from research prototypes. Therefore, research is needed to map the types of gamification available and improve the possible external validity as well [16,17,23]. In order to better grasp the phenomenon of gamification and its influence on peoples’ health behavior through mHealth apps in practice, we currently lack knowledge about how gamification is actually implemented in real-world mHealth apps and whether certain industry best practices have emerged. From our point of view, research and practice will benefit from such knowledge for various reasons. First, in conjunction with conceptual design knowledge, such deeper knowledge on gamification approaches in pertinent, real-world mHealth apps can guide developers in designing and implementing suitable gamification approaches with regard to their targeted health behavior and aid them in unleashing the full motivational potential of gamified mHealth apps. Second, for researchers in the field of gamified mHealth apps, such knowledge can serve as an indicator that shows whether, and if so, how extant research insights into the design of effective gamification in mHealth apps have been transferred to real-world systems. Third, such research contributes to a deeper understanding of the interplay between the effectiveness of gamification and its application context because it helps to get a better picture of whether certain gamification approaches have shown to be particularly suitable for certain health behaviors in practice. Providing practitioners and researchers with such knowledge requires scrutinizing the status quo of and understanding which, if any, dominant gamification approaches are being designed and implemented in mHealth apps. We therefore ask:

(Research Question) *What are dominant gamification approaches in mHealth apps?*

In literature, several studies exist that apply gamification to mHealth apps. However, most studies investigate the psychological or behavioral effects that occur when introducing specific game design elements (eg, points, leaderboards) or a combination of different elements to a certain mHealth app [24]. The stream of research that is closest to our work has been concerned with investigating the extent to which game design elements have been implemented in real-world mHealth apps and analyzing their relationships to various other constructs such as app popularity [15], user ratings [25,26], or the use of health behavior theory constructs [15]. To the best of our knowledge, no research exists that has explicitly examined the emergence of specific dominant gamification approaches in the domain of mHealth apps and the relationship to their targeted health behaviors.

In order to answer our research question, we draw on the taxonomy of gamification approaches in mHealth apps proposed by Schmidt-Kraepelin et al [20]. The taxonomy enables us to classify gamification approaches employed in pertinent gamified mHealth apps from the Apple App Store and Google Play Store. In addition, we use the 2-step clustering approach of Punj and Stewart [27], which has been employed in similar research [28,29], for identifying archetypes of gamification approaches in pertinent mHealth apps. In doing so, we are able to unveil established best practices in the design and implementation of gamification for mHealth apps and to analyze to what extent those gamification approaches are influenced by the underlying desired health-related outcomes.

## Methods

### Overview

**Figure 1:**
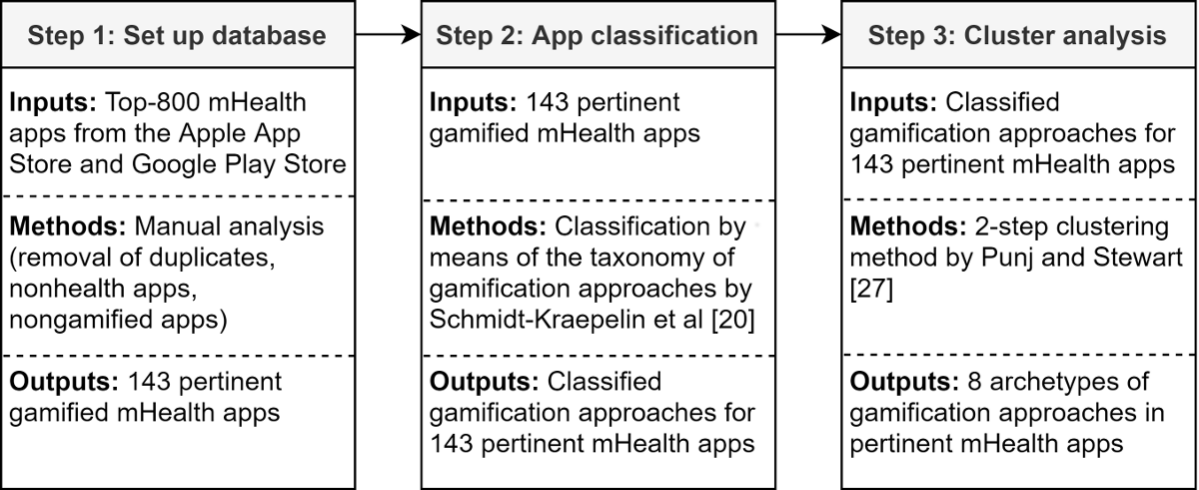
Overview of the 3-step research approach.

To answer our research question, we employed a 3-step research approach that was informed by the studies of Remane et al [28] and Thiebes et al [29] and is shown in Figure 1. As a first step, we set up a database of pertinent gamified mHealth apps from the Apple App Store and Google Play Store. Second, we classified the gamification approaches of the gamified mHealth apps within our sample based on the taxonomy provided by Schmidt-Kraepelin et al [20]. Finally, we conducted a 2-step cluster analysis in order to identify archetypes of the most dominant gamification approaches in pertinent gamified mHealth apps.

### Step 1: Setting up a Database of Pertinent Gamified mHealth Apps

**Figure 2:**
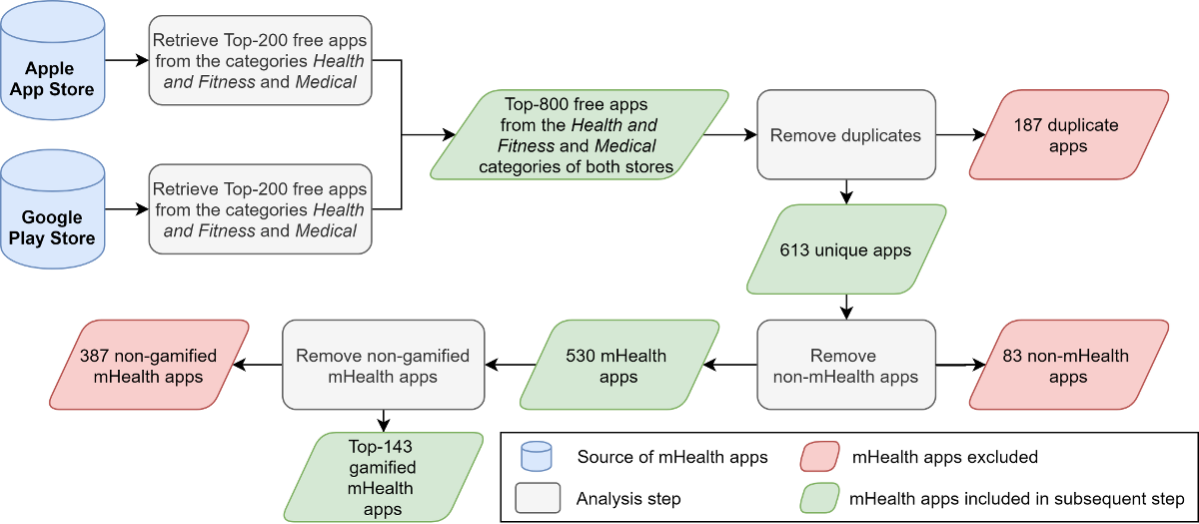
Setting up a database of pertinent gamified mHealth apps.

In the first step of this study, we set up a database of pertinent gamified mHealth apps for the 2 prevailing mobile operating systems: iOS and Android. For this purpose, we decided to sample mHealth apps from the Apple App Store and the Google Play Store because they are by far the 2 largest mobile app stores in terms of app downloads (Apple App Store: 29.6 billion in 2018; Google Play Store: 75.5 billion in 2018) and revenue (Apple App Store: US $46.6 billion in 2018; Google Play Store: US $24.8 billion in 2018) [30,31]. mHealth apps are usually found in the categories *Health and Fitness* and *Medical*, which exist on both the Apple App Store and the Google Play Store [32]. Both categories offer separate rankings, listing the top apps in terms of downloads for paid and free apps. As about 95% of apps downloaded to mobile devices are free and more than 95% of revenue in mobile app stores is generated through freemium apps (ie, apps that offer basic functionalities for free, but charge money for additional features) [33], we decided to consider only free apps within this study. Because the top rankings tend to change slightly from day to day, all rankings were recorded on January 18, 2018. To ensure a representative sample of mHealth apps, we exported the top 200 apps for both categories in both app stores. We decided to concentrate our analysis on the most popular apps since they hold the best chance of having successfully employed gamification because users tend to download and use them the most. This approach led to a set of 800 potentially relevant apps. If an app was recorded multiple times in the initial database (ie, once for the Apple App Store and once for the Google Play Store) and both versions offered identical functionality, one version of the app was excluded from further analysis. Overall, 187 duplicates were excluded. Furthermore, we examined all apps closely in order to determine (1) whether they were mHealth apps and (2) whether they were gamified (ie, made use of gamification). We excluded all apps from analysis that did not meet both requirements. To determine whether an app was an mHealth app, we followed the suggestions proposed by Stepanovic and Mettler [11]. Accordingly, an app had to either support patients in the treatment of a given medical condition (eg, diabetes), support users in pursuing healthy lifestyles (eg, weight control), or support medical professionals in the patient treatment or education. Overall, 530 apps were classified as mHealth apps. In a subsequent step, an app was considered as gamified, if it used gamification mechanics such as game-like rewards or incentives to increase motivation and sustain habits of users over time. As a reference point for gamification mechanics, the list proposed by Thiebes et al [22] was used. These game-like rewards and incentives had to support some underlying health-related functionality. For example, quizzes were only considered if their purpose was to motivate users to conduct the main health-related activity of the app. In this step, 143 of the remaining 530 apps were coded as gamified apps which constituted our final set of pertinent gamified mHealth apps. A complete list of apps included in and excluded from our database can be found in Multimedia Appendix 1, whereas an overview of the database setup process is given in Figure 2.

### Step 2: Classifying Gamification Approaches in mHealth Apps

For deriving meaningful clusters of gamification approaches, we needed a classification scheme to determine their characteristics. One type of such classification schemes are taxonomies, which serve as important tools for structuring knowledge in many scientific disciplines. Extant literature has already proposed taxonomies related to gamification. However, they often do not distinguish the concepts of gaming or serious games and gamification (eg, [34]), or only categorize single game design elements but not holistic gamification approaches (eg, [22]). In addition, many classification schemes consist of a set of dimensions (eg, game design elements) without providing concrete characteristics (ie, manifestations of these dimensions). Such taxonomies often leave too much room for interpretation, which impedes classifying real-world objects. In this work, we draw on the taxonomy proposed by Schmidt-Kraepelin et al [20] as our classification scheme, because it (1) provides the opportunity of classifying holistic gamification approaches as it is not limited to single game elements, (2) has been explicitly built for health care apps, and (3) provides a concrete set of dimensions with mutually exclusive and collectively exhaustive characteristics. Further, the taxonomy has been derived based on the guidelines proposed by Nickerson et al [35], which combine inductive and deductive reasoning and have been extensively used to develop taxonomies for phenomena in health care [29]. Past research has shown that taxonomies developed based on the guidelines by Nickerson et al [35] are suitable classification schemes with regard to the development of archetypes through cluster analysis [28,29]. The taxonomy proposed by Schmidt-Kraepelin et al [20] consists of 12 dimensions, each consisting of 2 to 3 mutually exclusive characteristics, with a total of 30 characteristics. The dimensions included in the taxonomy are (1) gamification concept-to-user communication, (2) user identity, (3) rewards, (4) competition, (5) target group, (6) collaboration, (7) goal setting, (8) narrative, (9) reinforcement, (10) level of integration, (11) persuasive intent, and (12) user advancement. The complete taxonomy is shown in Table 1. A detailed description of the taxonomy can be found in Multimedia Appendix 2.

To classify the apps in our database along the taxonomy, each app was downloaded, tested, and used to experience all features. Depending on the complexity of the mHealth app, the required analysis time was usually 15 to 30 minutes. Each mHealth app was coded by one of two researchers (MS-K and PT). Prior to data analysis, the researchers were trained in the understanding of the taxonomy by the authors who originally developed the taxonomy. In order to ensure a high level of coding reliability, both researchers coded an initial set of 20 gamified mHealth apps independently and subsequently discussed their results. This initial coding and the subsequent discussion were supervised by the original authors of the taxonomy. Afterward, the two researchers coded their assigned set of mHealth apps on their own. In cases where a researcher was uncertain about a coding, the respective gamified mHealth app was discussed with the other researcher and the authors of the original taxonomy. Final classifications for all 143 mHealth apps can be found in Multimedia Appendix 3.

**Table 1:** Taxonomy of gamification approaches for health apps proposed by Schmidt-Kraepelin et al [20].

Dimension	Rationale	Characteristics
Gamification concept-to-user communication	How does the gamification approach communicate with the user?	Direct Mediated
User identity	How is the user’s identity represented in the gamification approach?	Virtual character Self-selected
Rewards	Which rewards can users earn by playing and progressing within the gamification approach?	Internal Internal and external No
Competition	How do users compete with each other within the gamification approach?	Direct Indirect No
Target Group	Who is the targeted audience of the gamification approach?	Patients Healthy individuals Health professionals
Collaboration	Which form of collaboration does the gamification approach offer?	Cooperative Supportive only No
Goal setting	Who sets goals within the gamification approach?	Self-set Externally set
Narrative	How does the gamification approach behave over time?	Continuous Episodical
Reinforcement	How does the gamification approach attempt to reinforce its users?	Positive Positive–negative
Persuasive intent	Which type of health-related change does the gamification approach aim to evoke?	Compliance change Behavior change Attitude change
Level of integration	To which extent is the gamification approach cohesively related to the underlying health-related activities?	Independent Inherent
User advancement	How does the gamification approach consider the overall user advancement?	Presentation only Progressive No

### Step 3: Cluster Analysis

In the third step of our methodology, we derived archetypes of gamification approaches for mHealth apps, utilizing cluster analysis. Cluster analysis is a process of finding distinct groups of objects (ie, clusters) in data for which the objects within 1 group are as similar as possible, and as dissimilar as possible from objects in the other groups based on a predetermined set of attributes [36]. Many different clustering methods exist and choosing the approach best suited for the present problem can be cumbersome and error prone. For example, the researcher has to consider what similarity or dissimilarity measure to choose and how many clusters to generate [27]. While in general, iterative partitioning algorithms, such as k-means, yield better performance than hierarchical clustering methods, they usually require defining a priori how many clusters the researcher wants to produce. To overcome the weaknesses of both approaches and increase clustering performance, we followed the 2-step approach proposed by Punj and Stewart [27]. In the first step, a hierarchical method is used to determine a preliminary solution, from which a candidate number of clusters can be deduced. In the second step, this candidate number is then used as a starting point in an iterative partitioning algorithm in order to arrange the included objects into their final cluster solution. Because the objects within this study (ie, gamification approaches) are classified through the application of a taxonomy that is similarly structured like the taxonomies of Remane et al [28] and Thiebes et al [29], we followed the clustering approaches of those studies and utilized Ward’s method for step 1 and the k-means algorithm for step 2. Both steps were conducted with IBM’s statistical analysis software SPSS Statistics version 25.0.

The dendrogram produced by Ward’s method indicated that the 1-7-, 11-,12-, or 13-cluster solutions would all be suitable candidate numbers of clusters. Reviewing the scree plot, with use of the elbow rule [37], the cluster solutions of size 6, 13, and 8 stood out to have the most explanatory power in this particular order (cf. Multimedia Appendix 4). Hence, we narrowed the search space to 6-, 8-, and 13-cluster solutions. Having determined the preliminary cluster solutions, we used k-means to derive our final cluster solution in the second stage. The 6- and 13-cluster solutions produced by k-means both comprised clusters with size 1 or 2, which impeded a meaningful interpretation of these solutions. In addition to the small cluster sizes, all 3 solutions showed low to no significance (ie, how relevant a certain characteristic is for the cluster solution) of the characteristics Mediated (dimension: *gamification concept-to-user communication*) and virtual character (dimension: *user identity*) in the clustering process (cf. Multimedia Appendix 5). This finding was not surprising as only 1 out of 143 and 3 out of 143 objects, respectively, showed these characteristics and therefore would only influence clustering slightly if at all. Further testing confirmed that the low relevance of these characteristics and hence their respective dimensions was present for all partitioning sizes ranging from 2 to 19 clusters.

Consequently, in order to achieve relevance of all dimensions in the clustering process and to increase the explanatory power of our results, we omitted the dimensions *gamification concept-to-user communication* and *user identity* with their respective characteristics from the clustering, thus leaving 10 dimensions and 26 characteristics. With these new limitations the dendrogram produced by Ward’s method pointed toward 6, 8, 10, and 16 as preferred cluster solutions. Again, examining the scree plot with the elbow rule indicated 6-, 11-, 13-, and 8-cluster solutions to have the most explanatory power in this particular order (cf. Multimedia Appendix 6). Therefore, we selected 6, 8, 10, and 11 as our preliminary cluster solutions. In contrast to the k-means clustering of all 12 dimensions, the results for the 10 dimensions painted a much clearer picture. Moreover, relevance of characteristics was greatly improved throughout all possible cluster solutions (cf. Multimedia Appendix 7), which strengthened us in our decision to omit the 2 irrelevant dimensions from analysis. Because the 10- and 11-cluster solutions had small clusters of size 4 and smaller, we disregarded them for further analysis. Manual inspection for explanatory power showed that while we were able to find meaningful interpretations for both, 6- and 8-cluster solutions, the 8-cluster solution represented a more detailed and fine-grained picture for the landscape of gamification approaches in mHealth apps. Therefore, we selected the 8-cluster solution as the most suitable one for this study and report it below.

## Results

### Archetypes of Gamification Approaches for mHealth Apps

**Figure 3:**
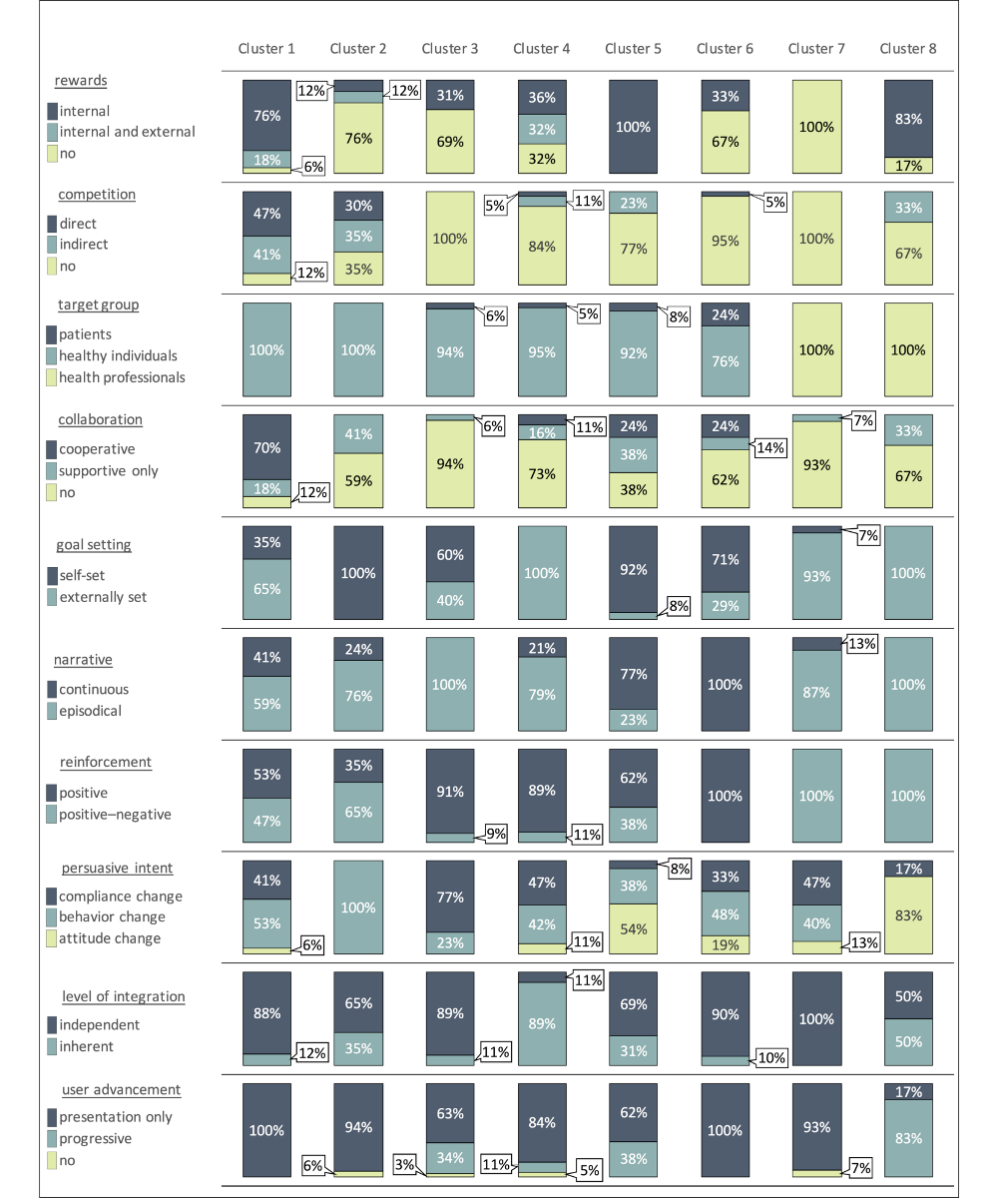
Cluster characteristics.

Each cluster of the 8-cluster solution contains 6-35 mHealth apps of the total 143 apps that incorporated gamification approaches from our database. Thereby, each cluster can be differentiated with regard to its most salient characteristics. Because the taxonomy development method by Nickerson et al [35] results in characteristics that are mutually exclusive and collectively exhaustive, the data can be interpreted as percentages [29]. A detailed overview of the results of the cluster analysis can be found in Multimedia Appendix 8, whereas Figure 3 illustrates the main characteristics of all clusters by the means of bar charts. For example, 24% (5/21) of apps in cluster 6 target patients, while 76% (16/21) have healthy individuals as their target group, and no apps (0%) were designed for health professionals. Based on the 8 identified clusters we derived 8 distinct archetypes of gamification approaches that are being implemented in mHealth apps. In the following, we elaborate on each archetype by highlighting its most representative characteristics, describing the most prevalent health behaviors that the archetype is used for and providing typical examples of mHealth apps for each archetype.

### Archetype 1: Competition and Collaboration

The first archetype is characterized by the fact that it is the only archetype that contains both competitive and collaborative gamification elements. It reinforces health behaviors with positive and negative motivational experiences. While the underlying health activity is mostly independent from the gamification approach, the *competition* and *collaboration* elements as well as internal *rewards* aim to help users in reaching externally and self-set goals, thus evoking compliance and behavioral change. Archetype 1 exclusively targets healthy individuals. It may draw on a continuous *narrative* but often relies on an episodical style. This archetype is primarily implemented in apps such as Sweatcoin Pays You To Get Fit, Pedometer, Step Counter & Weight Loss, or Fitbit. The targeted health behavior of these apps is physical activity and fitness. Consequently, they are primarily found in the category *Health and Fitness.* A typical example that implements archetype 1 is Nike+. This app offers daily and monthly running challenges in which users may participate and compete against each other while also providing elements of cooperative collaboration.

### Archetype 2: Pursuing Self-Set Goals Without Rewards

A unique characteristic of archetype 2 is that it exclusively draws on goals that are set by the users themselves. Furthermore, archetype 2 does not offer any type of explicit *rewards* for specific health-related activities (eg, badges, vouchers) to their users. Users usually do not have the opportunity for cooperative *collaboration,* but may be motivated through supportive *collaboration* (eg, connection to social media) and various forms of *competition*, which results in possibilities for positive and negative *reinforcement*. While the *narrative* is usually episodical, *user advancement* within the archetype is only presentational (eg, through consecutive user levels), but not progressive (eg, through unlocking new content or progressive levels of difficulty). Similar to archetype 1, archetype 2 is mainly implemented in apps that target physical activity, fitness, and nutrition. Apps such as Freeletics Bodyweight, Planet Tracker, and Fooducate Healthy Weight Loss & Calorie Counter are typical examples that implement archetype 2. Another example is the app Runtastic Running & Fitness Tracker. It allows users to track runs or other fitness activities and share them with others. Users may also send a “digital cheer” through the app to motivate peers while they are physically active.

### Archetype 3: Episodical Compliance Tracking

Archetype 3 typically does not draw on negative *reinforcement* or any type of social component such as *competition* or *collaboration*. An important characteristic of this archetype is that the narrative is always episodical, which means that it is clearly divided into different stages or that the user progress is partially or fully reset after a certain amount of time. Within these episodes, the *user advancement* may be presentational or progressive. Moreover, archetype 3 in some cases may also offer internal *rewards*. The main purpose of most apps that implement archetype 3 is to support healthy individuals in staying compliant to specific rules or guidelines for a certain period. Targeted health behaviors of archetype 3 are broad and include physical activity, fitness, an overall healthy lifestyle, nutrition, female health, pregnancy, meditation, mental health, and therapy adherence. Archetype 3 is implemented in apps such as BetterMe: Weight Loss Plan, Lifesum: Calorie Counter, Food & Nutrition Tracker, and Water Drink Reminder. The app Pregnancy Week by Week also implements archetype 3. It provides parents with insights into their baby’s development during pregnancy and gives advice on nutrition, exercise, and medical needs of the current week.

### Archetype 4: Inherent Gamification for External Goals

Archetype 4 uses gamification to help healthy individuals to meet externally set health goals. It mainly aims to foster a compliance or behavioral change within the users by drawing on internal or external *rewards*. Archetype 4 does not contain competitive or collaborative elements, but rather relies on positive *reinforcement*. It aims to improve the users’ health through an episodic *narrative* of externally set goals. Archetype 4 only has a presentational *user advancement*. A unique characteristic of this archetype is that its gamification elements are inherently connected to the mHealth apps themselves (ie, the health-related activity is partially or fully embedded in the gamification approach and cannot be performed without interacting with the gamification approach). Similar to archetype 3, the targeted health behaviors of apps that implement archetype 4 are rather broad. They include physical activity, fitness, meditation, mental health, health navigation (eg, directories of physicians), and therapy adherence. Typical example apps are Ada – Your Health Companion, 30 Day Fit Challenge Workout, and Map My Fitness Workout Trainer. Another example is Moodpath. This app provides users with a mood diary, which helps them to better understand their feelings and thoughts with episodical interactive sessions on topics such as depressions, sadness, and anxiety.

### Archetype 5: Internal Rewards for Self-Set Goals

Archetype 5 utilizes gamification in an attempt to evoke a change in the users’ behavior or attitude. This is achieved by omitting elements of *competition,* but providing users with various forms of *collaboration*. A unique characteristic of this archetype is that it solely draws on internal *rewards* (eg, points, badges). Users set goals themselves and may experience positive and negative *reinforcement* as a result of their decisions. Many apps that implement archetype 5 focus on supporting meditation and mental health. However, it is also used to support female health, pregnancy, and therapy adherence. Typical examples are apps such as Smoke Free - Quit Smoking Now, and Aura: Calm Anxiety & Stress Chat. The app Headspace: Guided Meditation offers a range of meditation programs revolving around different life aspects such as Foundation, Sport, Health, Relationships, or Performance. Within each session the user may learn new meditation techniques and strategies to improve their mental well-being.

### Archetype 6: Continuous Assistance Through Positive Reinforcement

Archetype 6 targets healthy individuals and patients that require continuous assistance with a certain health issue. It offers no *rewards* and does not provide progressive *user advancement*. In addition, it does not incorporate any competitive elements and *reinforcement* is only positive. While this archetype is not limited to a specific *persuasive intent* or *goal setting*, the *level of integration* is primarily independent. Overall, archetype 6 is in many aspects similar to archetype 3. However, the main difference is that archetype 6 draws on a continuous *narrative* in order to meet users’ needs for continuous assistance. The targeted health behaviors of apps that implement archetype 6 include physical activity, fitness, nutrition, female health, pregnancy, and therapy adherence. Typical examples of such apps are mySugr: the blood sugar tracker made just for you, DreamMapper, and Kindara: Fertility Tracker. The app Pill Reminder supports users in tracking their medication with the help of notifications and a cooperative function that grants caretakers insight into the medication adherence of the patient.

### Archetype 7: Positive and Negative Reinforcement Without Rewards

In contrast to the archetypes described so far, archetype 7 solely targets health professionals. It does not provide any *competition*, *collaboration*, or *rewards* and goals are set externally. While archetype 7 implies positive and negative *reinforcement* strategies, *user advancement* is only presentational and not progressive. Archetype 7 is only implemented in mHealth apps that support medical education of health professionals such as Teach Me Anatomy, NCLEX-RN Pocket Prep, or EMT-B Pocket Prep. Another typical example is the app ATI TEAS Pocket Prep. This app aims to provide users with necessary knowledge and skills through a simple knowledge database and a classical quiz structure.

### Archetype 8: Progressive Gamification for Health Professionals

Archetype 8 is similar to archetype 7 in the sense that it solely targets health professionals. However, these 2 archetypes also differ to some extent. The most prominent difference is that archetype 8 primarily draws on a progressive *user advancement*, which means that either new content may be progressively unlocked or levels of difficulty may increase while users deepen their knowledge and skills. In contrast to archetype 7, archetype 8 is also utilized as a means to change the attitude of users. Similar to archetype 7, archetype 8 is solely implemented in mHealth apps that support medical education of health professionals. Apps that implement this archetype include Airway Ex, Prognosis: Your Diagnosis, or ATI TEAS 6 Practice Test. The app Touch Surgery is another typical example. It lets students compete against each other indirectly by ranking how well they performed a surgery case within the mHealth app and gives them the possibility to communicate and rate surgery cases of each other.

## Discussion

### Principal Results

Overall, the findings of our study help to better grasp the phenomenon of gamification and its influence on peoples’ health behavior through mHealth apps in real-world systems. Analysis of our derived archetypes reveals some interesting insights into the current landscape of gamification approaches that are being utilized in pertinent mHealth apps. Our results paint a heterogeneous landscape of different gamification approaches for mHealth apps and help to explain the relationship between industry best practices for gamification and targeted health behaviors. The results also indicate that gamification approaches in real-world mHealth apps differ in some aspects from gamification approaches that are deployed in research prototypes, which underlines the value of consistently contrasting conceptual knowledge against real-world observations. The main findings of this study are discussed in the following.

First, our results give insight into the variety of different gamification approaches and paint a heterogenous landscape of different gamification approaches for mHealth apps. While some archetypes seem closer to classical notions of gamification in health (eg, archetype 1), other approaches have received substantially less attention in extant research. Overall, our results highlight the richness of opportunities and different perspectives that gamification provides when it comes to augmenting mHealth apps with motivational affordances. Gamification cannot only be applied with regard to a lot of different health-related outcomes, but it can also come in various forms and shapes.

Second, our results also indicate a close relationship between the identified archetypes of gamification approaches in mHealth apps and the actual health behavior change that is being targeted by such mHealth apps. We further analyzed this finding by investigating the relationship between the most significant archetype characteristics and the targeted health behavior. The results of this additional analysis, in general, support our findings (cf. Multimedia Appendices 9 and 10). It is noticeable that certain archetypes of gamification approaches are in practice primarily used for certain health behavior changes. For developers of gamified mHealth apps these insights provide valuable points of reference for implementing gamification that targets to support a specific health behavior. For example, when it comes to getting people to live a more active life and increase their physical activity, our results indicate that archetype 1 and archetype 2 are implemented frequently. These gamification approaches are characterized by a high degree of competition, which could be inappropriate in other, more serious, health contexts and could even lead to negative effects (eg, demoralization of users due to overemphasizing of peer pressure) [38]. The two archetypes differ in particular with regard to the dimension of *goal setting* (ie, whether goals are set by the user or determined externally) and *collaboration* (ie, whether collaboration is cooperative, supportive only, or no possibility for collaboration is implemented). A completely different form of gamification approaches, however, is used in apps that are intended to support future health professionals in their training. These typically implement archetype 7 and archetype 8. They are characterized by the fact that the goals to be achieved are set externally and the *narrative* is episodic. However, both archetypes are also fundamentally different in some characteristics. For example, while archetype 7 does not offer any type of *rewards* and draws only on presentational *user advancement*, archetype 8 aims to motivate their users with internal *reward*s and adjust difficulty levels in a progressive *user advancement*. In contrast to the previously discussed gamification approaches for fitness apps, archetype 7 and archetype 8 do not provide any form of *competition*. This could be an expression of the fact that the use of *competition* in learning environments is often seen as problematic and controversial in research [39]. On the one hand, researchers report that *competition* is used in classrooms to draw the attention of learners and motivate learning [39,40]. On the other hand, researchers have often raised concerns that incautious implementations of *competition* in educational settings may create anxiety and impede performance [41]. Also, other clusters indicate a close relationship between the targeted health behavior and the implemented gamification approach. For example, when it comes to support mental health activities (archetype 5), developers of gamified mHealth apps primarily avoid using gamification that potentially puts additional pressure on users (eg, competition, externally set goals) and instead aim to foster positive experiences by allowing self-set goals and *collaboratio*n. For health behaviors that require continuous assistance (archetype 6: eg, chronic disease management, medication adherence), especially continuous narratives seem to be a popular approach to meet users’ motivational needs. By contrast, developers of mHealth apps that implement this archetype primarily do not use elements of *competition* or any type of *rewards*. Contrasting the previously discussed archetypes, the range of targeted health behaviors for archetype 3 and archetype 4 is substantially broader. Instead of being tightly related to a specific health behavior, these archetypes seem to be applicable for various different health behaviors. For example, archetype 3 uses episodic narratives in order to motivate users to stay compliant with a temporary health behavior (eg, following pregnancy guidelines). By contrast, apps that implement archetype 4 draw on gamification approaches that are inherently related to the targeted health behavior in order to motivate users to achieve externally set goals (eg, completing a specific 30 days fitness challenge).

Third, the manual analysis and interpretation of our 8-cluster solution indicates that archetypes of gamification approaches for mHealth apps seem to be less clearly definable in comparison to other use cases of this method (in this regard, we particularly refer to the studies by Thiebes et al [29] and Remane et al [28]). This observation is also supported by statistical indicators. For example, in our study of gamification approaches for mHealth apps the analysis of the dendrogram resulted in considerably more cluster sizes as suitable candidates than the dendrogram in the study by Thiebes et al [29]. A similar picture was drawn when applying the elbow rule (ie, comparing the slopes of cluster size candidates within the scree plots), where the 6-cluster solution was clearly identified as the dominating solution by Thiebes et al [29], while we were left with multiple possible solutions that performed relatively equal with regard to statistical measures. From our point of view, there are different explanations for this observation. For example, it might be the case that our taxonomy of gamification approaches allows more meaningful combinations of characteristics than the taxonomies for business models proposed by Thiebes et al [29] and Remane et al [28], which leads to a more heterogeneous landscape and less clearly separable archetypes. Furthermore, it should also be noted that the development and provision of mHealth apps in most cases require significantly less capital and economic risk taking than direct-to-consumer genetic testing services [29] or carsharing services [28]. As a consequence, providers of gamified mHealth apps may simply be more willing to experiment around with their gamification approaches and do not necessarily have to draw on best practices. Lastly, the concept of gamification is still rather new and has just recently become increasingly popular for mHealth apps [24]. Thus, best practices for gamification approaches in mHealth apps might still be in an emerging phase and become clearer in the future.

Fourth, when comparing our classification results with the classification results that emerged during the taxonomy development process [20], large differences between theoretical considerations on gamification in mHealth apps and their implementation in practice became apparent. These differences become particularly clear in the dimensions *gamification concept-to-user communication* and *user identity*. During the taxonomy development process, in which exclusively 27 mHealth apps presented in research papers (primarily research prototypes) were examined, 5 of 27 apps were classified with a mediated *gamification concept-to-user communication* and 6 of 27 apps were classified with a virtual character as the characteristic for *user identity*. By contrast, our classification of a total of 143 pertinent mHealth apps offered in the Apple App Store and Google Play Store showed only 3 apps with a mediated *gamification concept-to-user-communication* and only 1 app with a virtual character as form of *user identity*. In the case of virtual characters, this may be explained to some extent by the consideration of user preferences by developers of mHealth apps. A recent study showed that avatars (the most common form of virtual characters in gamification approaches) are among those game design elements that are least preferred by users of mHealth apps [42]. However, existing research has shown that the successful use of avatars in gamification approaches is complex and requires a profound consideration of individual user needs [43]. Only by incorporating these individual needs and avoiding one-size-fits-all solutions, a desired level of emotional attachment and extraneous cognitive load may be achieved [43]. Our results could be interpreted as an indicator that most developers of mHealth apps are not willing to put this effort into the development of virtual characters, especially because users seem to attach less importance to their inclusion in comparison to other game design elements.

### Implications

Our study yields several implications for research and practice. For research, our work contributes to a more comprehensive understanding of mHealth apps. We utilized a systematic classification of gamification approaches in order to develop archetypes that have emerged in pertinent mHealth apps. In doing so, we strengthen the previously formulated notion [28,29] that combining classifications based on the guidelines by Nickerson et al [35] with cluster analysis methods such as Ward’s method and k-means is a promising approach to uncover archetypes in emerging contexts. Furthermore, our research demonstrates that the taxonomy proposed by Schmidt-Kraepelin et al [20] is applicable not only for research prototypes but also for real-world systems. However, our results also indicate that some dimensions and characteristics (eg, direct *gamification concept-to-user communication* and virtual characters as *user identity*) seem to be much less relevant in practice than previously assumed. Compared with other archetypes of mHealth apps that have been proposed in the literature [44], the archetypes presented here focus on a rather narrow aspect of mHealth apps (ie, their use of gamification for motivational purposes). This allowed us to clearly state how gamification has predominantly been instantiated in mHealth apps. In particular, our results indicate that specific best practices for gamification approaches have emerged for certain targeted health behavior changes. Hereby, our research contributes to answering the call for more research that helps to select suitable persuasive architectures for different mHealth apps [9].

For practice, our research yields important implications for providers and developers of mHealth apps, who want to utilize gamification in order to motivate desired health behaviors. The presented archetypes provide these stakeholders with blueprints of potential gamification approaches that have been utilized in pertinent mHealth apps. In particular, when regarding the close connection between gamification approach archetypes and underlying health behaviors (eg, physical activity, education of health professionals, mental health guidance), such blueprints can be used as guidance for designing suitable gamification approaches. Although our work does not account for the efficiency of these archetypes with regard to motivational or health behavior outcomes, it does provide practitioners with best practices that have been established among the most popular mHealth apps. Our results thereby are in line with the most prominent gamification design frameworks that emphasize the importance of specifying the targeted (health) behavior before designing a gamification approach [18]. We would also like to stress that our provided blueprints cannot substitute following established design frameworks. Rather, they should be used as an additional means to triangulate the results of following such design frameworks. Overall, our work contributes to a better understanding of how gamification is being applied in real-world mHealth apps. Such knowledge becomes increasingly important given that more and more apps draw on motivational techniques in order to achieve a desired level of user motivation and stand out from the mass of available mHealth apps.

### Limitations and Future Research

The findings of this study should be interpreted in consideration of some key limitations. First, gamification is a relatively young and constantly evolving phenomenon that just recently started to draw increasing attention by researchers and practitioners alike. Disregarding this aspect, our archetypes represent only a snapshot of the current landscape of gamification approaches in pertinent mHealth apps. It is likely that in the near future, new insights from research and practice may guide the development of innovative and more effective gamification approaches (eg, through stronger consideration of individual and contextual factors that determine the effectiveness of gamification). Future research may answer this limitation by closely examining the evolution of knowledge on effective gamification approaches in mHealth apps and how they are designed. Second, we limited our analysis to free mHealth apps on the Apple App Store and Google Play Store which might have led to the exclusion of relevant paid apps or mHealth apps from other app stores. However, it would have been complicated and costly to deal with the different revenue and pricing models of freemium, premium, and others. With an industry trend toward free mobile apps [15], 95% of downloaded apps being free, and more than 95% of revenue in mobile app stores being generated through freemium business models [33], we are confident that this sample inherits the majority of gamification approaches used in mHealth apps today. Furthermore, we think that the categorical decision of only including free apps from the Apple App Store and Google Play Store ensures the reproducibility of the results. Nonetheless, investigating paid apps or alternative app stores could potentially reveal further insights. Third, the sample of 143 gamification approaches is notably smaller than that of similar studies in other contexts (eg, the 277 direct-to-consumer genetic testing services investigated by Thiebes et al [29]). This may have led to some clusters being underrepresented in our analysis. In addition, we focus our analysis on the most popular mHealth apps only, because our goal was to identify the best practices of gamification approaches. However, this certainly limits the explanatory power of our results as mHealth apps that have a very small and specific target group (eg, people with very specific and rare diseases) and still implement valuable gamification approaches may be underrepresented in our sample. It may be an interesting avenue for future research to assess whether less popular apps utilize gamification in a different way. In this regard, additional measures for mHealth app popularity (eg, user rating or number of users) could be taken into account in order to include more niche mHealth apps in the analysis. Such research could additionally contribute to the upcoming research stream that aims to assess the potential effect that gamification may have on mHealth app success [25]. Fourth, the coding of each mHealth app by only 1 researcher might have led to false classification in terms of the gamification approaches, which then could have impacted the cluster analysis. However, both researchers were trained prior to the coding to have an identical understanding of the gamification approaches confined within the taxonomy in order to minimize human error in this process. Furthermore, with larger groups of objects the cluster analysis tends to be less sensitive toward outliers, which decreases the effects of false coding. Additionally, our approach included 2 different clustering methods (ie, Ward’s Method and k-means) to further minimize the risk of creating inaccurate cluster solutions due to outliers. Finally, it must be noted that we omitted the 2 dimensions *gamification concept-to-user communication* and *user identity* when conducting the cluster analysis. However, we are confident that this was the best possible approach to achieve meaningful results because omitting both dimensions during the analysis resulted in substantially higher explanatory power of the clusters and only a negligible number of objects (of the 143 apps analyzed, only 1 showed the characteristic virtual character in the dimension *user identity* and only 3 showed the characteristic mediated in the dimension gamification *concept-to-user communication* showed the inferior characteristics.

### Conclusions

Gamification is a relatively young and constantly evolving phenomenon that is increasingly being utilized in the health care sector and thus becomes more and more important for researchers, health professionals, and providers of digital health services. In this study, we propose 8 rigorously developed archetypes of gamification approaches that illustrate how gamification is being implemented in mHealth apps and how their design is determined by the targeted health behavior. In doing so, we unveil salient best practices, and thereby contribute to a more profound understanding of gamification in mHealth apps. Our results can serve as a foundation for future research that advances our knowledge on how gamification may positively influence health behavior change and guide practitioners in the design and development of highly motivating and effective mHealth apps.

## Appendix

Multimedia Appendix 1 Overview of included and excluded mHealth apps.

Multimedia Appendix 2 Detailed description of taxonomy proposed by Schmidt-Kraepelin et al [20].

Multimedia Appendix 3 Taxonomy coding overview.

Multimedia Appendix 4 Cluster analysis details Ward’s Method 12 dimensions.

Multimedia Appendix 5 Cluster analysis details k-means method 12 dimensions.

Multimedia Appendix 6 Cluster analysis details Ward’s Method 10 dimensions.

Multimedia Appendix 7 Cluster analysis details k-means method 10 dimensions.

Multimedia Appendix 8 Overview of clusters.

Multimedia Appendix 9 Relationship between archetype characteristics and targeted health behavior.

Multimedia Appendix 10 Targeted health behaviors per archetypes.
