# Supplementary material for: Archetypes of Gamification: Analysis of mHealth Apps
Source: JMIR Mhealth Uhealth. 2020 Oct 19;8(10):e19280. doi: 10.2196/19280 (PMC7605978; doi:10.2196/19280)
Supplement: Multimedia Appendix 2 [file mhealth_v8i10e19280_app2.docx]

## Multimedia Appendix 2. Detailed description of taxonomy proposed by Schmidt-Kraepelin et al [1].

The taxonomy proposed by Schmidt-Kraepelin et al [1] consists of 12 dimensions, each consisting of two to three mutually exclusive characteristics, with a total of 30 characteristics. The dimensions included in the taxonomy are *(1) gamification concept-to-user communication*, *(2) user identity*, *(3) rewards*, *(4) competition*, *(5) target group*, *(6) collaboration*, *(7) goal-setting*, *(8) narrative*, *(9) reinforcement*, *(10) level of integration*, *(11) persuasive intent*, and *(12) user advancement*. As the taxonomy was developed based on the rigorous guidelines proposed by Nickerson et al [2], its characteristics are mutually exclusive (ie, no object can be assigned to two different characteristics in a dimension) and collectively exhaustive (ie, each object must be assigned to one of the characteristics in a dimension). Table MA2-1 gives an overview of the taxonomy’s dimensions and characteristics. In addition, the taxonomy is explained in more detail in the following.

Table MA2-1. Taxonomy of gamification approaches for health apps proposed by Schmidt-Kraepelin et al [1].

| Dimension | Characteristic |
| --- | --- |
| Gamification concept-to-user communication | Direct; Mediated |
| User identity | Virtual character; Self-selected |
| Rewards | Internal; Internal and external; No |
| Competition | Direct; Indirect; No |
| Target group | Patients; Healthy individuals; Health professionals |
| Collaboration | Cooperative; Supportive only; No |
| Goal-setting | Self-set; Externally set |
| Narrative | Continuous; Episodical |
| Reinforcement | Positive; Positive-negative |
| Persuasive intent | Compliance change; Behavior change; Attitude change |
| Level of integration | Independent; Inherent |
| User advancement | Presentation only; Progressive; No |

### Gamification Concept-to-User Communication

How does the gamification approach communicate with the user? The gamification approach can either deliver messages directly to the user utilizing textual or audio outputs (*direct*) or by a mediator interacting with the user such as an avatar (*mediated*).

### User identity

How is the identity of the user represented in the gamification approach? On the one hand, gamification approaches may allow users to choose a static self-selected identity such as a combination of a nickname and a picture or similar elements (*self-selected*). On the other hand, gamification approaches may offer the feature to create a unique virtual character, such as avatars, which can be customized over time representing the progression of the user (*virtual character*).

### Rewards

Which rewards can users earn by playing and progressing within the gamification approach? Gamification approaches offer three kinds of rewards. First, virtual rewards that hold no real-world value and are only accessible within the gamification approach (*internal*) such as badges and achievements. Second, in addition to internal rewards, gamification approaches may also include real-world (*external*) rewards such as discounts on physical purchases. Lastly, other gamification approaches may not offer any rewards at all (*no*).

### Competition

How do users compete with each other within the gamification approach? There are three kinds of competition. The first is direct competition, where users can compete against each other on the same specific task (*direct*). Both users take on the same challenge or task and may then compare their performances. The second kind is indirect competition (*indirect*). In such gamification approaches, users only indirectly compare their overall performance with all other users of the application (eg, through point systems and leaderboards). Lastly, some gamification approaches do not offer any form of competition between users (*no*).

### Target Group

Who is the targeted audience of the gamification approach? Specifically, mHealth apps can have three target groups, namely patients (*patients*), healthy individuals (*healthy individuals*) or health professionals (*health professionals*). It is important to differentiate the target group of the mHealth app and the target group of the gamification approach. Even though both target groups may be identical, the target group for the gamification approach might only be a subgroup of the target group for the mHealth app. An example for this case is an mHealth app that allows health professionals to provide content for their patients, but the gamification approach only aims to motivate patients to use the system.

### Collaboration

Which form of collaboration does the gamification approach offer? Some gamification approaches allow users to actively cooperate on a specific task or challenge, thus cooperatively contributing to the accomplishment of the task (*cooperative*). Other gamification approaches only offer supportive collaboration (*supportive only*). In this approach users can be motivated by other people that do not necessarily need to be users of the app, (eg, support via social networks). Lastly, some gamification approaches do not offer any form of collaboration (*no*).

### Goal-Setting

Who sets goals within the gamification approach? Some gamification approaches allow the user to set their own goals (*self-set*), either completely free or by selecting goals from a predefined list of goals provided by the gamification approach. Other gamification approaches come with externally set goals (*externally set*). In such gamification approaches, goals are set by external stakeholders such as the app developers or a health professional.

### Narrative

How does the gamification approach behave over time? The gamification approach can be episodical, meaning the gamification approach is clearly divided into different stages (*episodical*). In episodical narratives, the user’s progress may also be reset partially or fully after a certain time. In contrast to the episodical approach the gamification approach can also be continuous. This means, that it is not divided into differentiable stages and the progress of the user is never reset (*continuous*).

### Reinforcement

How does the gamification approach attempt to reinforce its users? Schmidt-Kraepelin et al [1] found that apps either only use positive reinforcement to motivate their users (*positive*), for example, by highlighting current and future successes, or additionally use negative reinforcement (*positive-negative*). Positive-negative reinforcement includes referring to failures from the past or reducing already earned points.

### Persuasive Intent

Which type of health-related change does the gamification approach aim to evoke? There are three types of persuasive intents that gamification approaches aim at. First, compliance change means that the gamification approach aims to make users compliant to very specific rules or guidelines in order to improve their health (*compliance change*). Second, behavioral change refers to gamification approaches that aims to foster health-promoting behavior in a specific context without strict rules or guidelines (*behavior change*). Lastly, attitude change means that the gamification approach is designed to influence the user’s fundamental attitude towards a certain health-related topic (*attitude change*).

### Level of Integration

To which extent is the gamification approach cohesively related to the underlying health-related activities? If the underlying health-related activities could be easily performed without the gamification approach and the gamification approach is superficially implemented, the level of integration is independent (*independent*). In contrast to this, if the health-related activities are partially or fully embedded into the gamification approach, it is inherent (*inherent*). An example for this would be dividing the activities into missions that are inherently connected to the gamification approach.

### User Advancement

How does the gamification approach consider the overall user advancement? The overall advancement of the user may either be in a presentational manner only, for example, through experience points or progress bars (*presentation only*). Adding to this, the gamification approach may also adopt to the users’ progress, for example, by allowing them to unlock higher difficulty levels when reaching a certain amount of experience points (*progressive*). Alternatively, some gamification approaches do not consider the users’ overall advancement at all (*no*).

## References

1. Schmidt-Kraepelin M, Thiebes S, Tran MC, Sunyaev A. What’s in the Game? Developing a Taxonomy of Gamification Concepts for Health Apps. Proceedings of the 51st Hawaii International Conference on System Sciences; 2018 Jan 3-6; Waikoloa, Hawaii, USA. Hawaii: University of Hawai'i at Manoa; 2018. p. 1217-26.

2. Nickerson RC, Varshney U, Muntermann J. A method for taxonomy development and its application in information systems. European Journal of Information Systems. 2013;22(3):336-59. doi:10.1057/ejis.2012.26.
