# Supplementary material for: Archetypes of Gamification: Analysis of mHealth Apps
Source: JMIR Mhealth Uhealth. 2020 Oct 19;8(10):e19280. doi: 10.2196/19280 (PMC7605978; doi:10.2196/19280)
Supplement: Multimedia Appendix 4 [file mhealth_v8i10e19280_app4.docx]

## Multimedia Appendix 4. Cluster analysis details Ward’s Method 12 dimensions.

Table MA4-1. Agglomeration Schedule (Excerpt).

| **Iteration^a^** | **# of Clusters^b^** | **Coefficient^c^** | **Slope^d^** | **Difference in Slope^e^** |
| --- | --- | --- | --- | --- |
| **…** | **…** | **…** | **…** | **…** |
| 123 | 20 | 242.258 | 8.433 | 0.890 |
| 124 | 19 | 250.901 | 8.643 | 0.210 |
| 125 | 18 | 259.801 | 8.900 | 0.257 |
| 126 | 17 | 269.210 | 9.409 | 0.509 |
| 127 | 16 | 278.652 | 9.443 | 0.034 |
| 128 | 15 | 288.219 | 9.567 | 0.124 |
| 129 | 14 | 298.052 | 9.833 | 0.267 |
| 130 | 13 | 308.532 | 10.479 | 0.646 |
| 131 | 12 | 321.004 | 12.472 | 1.992 |
| 132 | 11 | 335.884 | 14.880 | 2.408 |
| 133 | 10 | 354.005 | 18.121 | 3.241 |
| 134 | 9 | 372.225 | 18.220 | 0.099 |
| 135 | 8 | 390.675 | 18.449 | 0.229 |
| 136 | 7 | 410.207 | 19.533 | 1.083 |
| 137 | 6 | 431.593 | 21.386 | 1.853 |
| 138 | 5 | 461.286 | 29.693 | 8.307 |
| 139 | 4 | 495.581 | 34.295 | 4.601 |
| 140 | 3 | 533.632 | 38.051 | 3.756 |
| 141 | 2 | 584.599 | 50.967 | 12.917 |
| 142 | 1 | 657.399 | 72.799 | 21.832 |

| a. Iteration of Ward’s Method. Since we have 143 objects (apps) we need 142 iterations to combine all objects into a single cluster.  b. Number of remaining Clusters. Each iteration the two closest of the remaining clusters are combined.  c. Error coefficient of combining two cluster. The more heterogenous the combined clusters are. the higher the error coefficient.  d. The slope is the total increase of the error coefficient between two iterations. A good cluster solution is found in the previous iteration of a sudden jump of the slope. avoiding the merging of two heterogenous clusters.  e. The difference in slope is the total increase of the slope between two iterations. A large increase of the difference in slope indicates a sudden jump of the slope. |
| --- |

Figure MA4-1. Scree Plot.

| Legend:  = Elbow  The scree plot aids in visual representation of the agglomeration schedule. It plots the iteration and the coefficient. Sudden jumps in the slope can now be identified with the elbow rule. An “elbow” is found where the slope previous to the “elbow” (ie. iteration) is low and the subsequent slope is high. |
| --- |
