# Supplementary material for: Archetypes of Gamification: Analysis of mHealth Apps
Source: JMIR Mhealth Uhealth. 2020 Oct 19;8(10):e19280. doi: 10.2196/19280 (PMC7605978; doi:10.2196/19280)
Supplement: Multimedia Appendix 7 [file mhealth_v8i10e19280_app7.docx]

## Multimedia Appendix 7. Cluster analysis details k-means method 10 dimensions.

Table MA7-1. k-means evaluation table.

| **Cluster solution** | | **5** | **6** | **7** | **8** | **9** | **10** | **11** | **13** |
| --- | --- | --- | --- | --- | --- | --- | --- | --- | --- |
| Number of iterations^a^ | | 10 | 6 | 6 | 6 | 5 | 9 | 9 | 6 |
| Theoretical Ø Cluster Size^b^ | | 29 | 24 | 21 | 18 | 16 | 15 | 13 | 11 |
| Small Clusters’ Size^c^ | | 17 | 17 | 7 | 6 | 7 | 3 | 4;4 | 3;4 |
| Number of clusters supported by dendrogram | | Yes | Yes | No | Yes | No | Yes | No | No |
| Number of clusters supported by elbow rule | | No | Yes | No | Yes | No | Yes | No | No |
| Number of characteristics with *Sig.* ≥ 0.001^d^ | | 7 | 8 | 6 | 3 | 6 | 3 | 3 | 1 |
| Dimension | Characteristic | *Sig.* | | | | | | | |
| Gamification concept-to-user communication | Direct | - | - | - | - | - | - | - | - |
|  | Mediated | - | - | - | - | - | - | - | - |
| User identity | Virtual character | - | - | - | - | - | - | - | - |
|  | Self-selected | - | - | - | - | - | - | - | - |
| Rewards | Internal | .000 | .000 | .000 | .000 | .000 | .000 | .000 | .000 |
|  | Internal and external | .000 | .033 | .003 | .000 | .000 | .000 | .000 | .000 |
|  | No | .000 | .000 | .000 | .000 | .000 | .000 | .000 | .000 |
| Competition | Direct | .000 | .000 | .001 | .000 | .000 | .000 | .000 | .007 |
|  | Indirect | .000 | .000 | .000 | .000 | .000 | .000 | .000 | .000 |
|  | No | .000 | .000 | .000 | .000 | .000 | .000 | .000 | .000 |
| Target group | Patients | .163 | .000 | .236 | .037 | .025 | .000 | .151 | .000 |
|  | Healthy individuals | .000 | .000 | .000 | .000 | .000 | .000 | .000 | .000 |
|  | Health professionals | .000 | .000 | .000 | .000 | .000 | .000 | .000 | .000 |
| Collaboration | Cooperative | .018 | .012 | .050 | .000 | .000 | .001 | .005 | .000 |
|  | Supportive only | .002 | .015 | .090 | .024 | .007 | .000 | .000 | .000 |
|  | No | .000 | .000 | .000 | .000 | .000 | .000 | .000 | .000 |
| Goal-setting | Self-set | .000 | .000 | .000 | .000 | .000 | .000 | .000 | .000 |
|  | Externally set | .000 | .000 | .000 | .000 | .000 | .000 | .000 | .000 |
| Narrative | Continuous | .000 | .000 | .000 | .000 | .000 | .000 | .000 | .000 |
|  | Episodical | .000 | .000 | .000 | .000 | .000 | .000 | .000 | .000 |
| Reinforcement | Positive | .000 | .000 | .000 | .000 | .000 | .000 | .000 | .000 |
|  | Positive-negative | .000 | .000 | .000 | .000 | .000 | .000 | .000 | .000 |
| Persuasive intent | Compliance | .000 | .004 | .000 | .000 | .000 | .010 | .010 | .000 |
|  | Behavior change | .000 | .000 | .000 | .000 | .000 | .000 | .000 | .000 |
|  | Attitude change | .069 | .001 | .000 | .000 | .001 | .000 | .000 | .000 |
| Level of integration | Independent | .005 | .000 | .000 | .000 | .007 | .000 | .000 | .000 |
|  | Inherent | .005 | .000 | .000 | .000 | .007 | .000 | .000 | .000 |
| User advancement | Presentation only | .000 | .015 | .000 | .000 | .000 | .000 | .000 | .000 |
|  | Progressive | .000 | .002 | .000 | .000 | .000 | .000 | .000 | .000 |
|  | No | .486 | .784 | .704 | .846 | .007 | .763 | .652 | .000 |

| a. Fewer iterations indicate more stable cluster partitions, as convergence is achieved quicker. b. Calculated by dividing n = 143 objects by the number of desired clusters k. This is the theoretical average size of each cluster.  c. Only showing clusters that are below 50% of the Theoretical Ø Cluster Size or the single smallest cluster size. Small clusters have less explanatory power/not enough objects to deduct meaningful archetypes. Nonetheless, a small cluster might just be underrepresented, and a larger sample size could allow meaningful interpretation, if the cluster increases. d. ANOVA results show significance values (*Sig.*) for each variable (ie, characteristic of the taxonomy) with 0 ≤ Sig. ≤ 1. A low *Sig.* indicates that the characteristic is relevant for the cluster solution. Thus, the optimal cluster solution should have few Sig. > 0. |
| --- |
