# Supplementary material for: Archetypes of Gamification: Analysis of mHealth Apps
Source: JMIR Mhealth Uhealth. 2020 Oct 19;8(10):e19280. doi: 10.2196/19280 (PMC7605978; doi:10.2196/19280)
Supplement: Multimedia Appendix 8 [file mhealth_v8i10e19280_app8.docx]

## Multimedia Appendix 8. Overview of clusters.

Table MA8-1. Crosstab analysis results.

| **Dimensions^a^** | **Characteristics^b^** | **Cluster 1** | **Cluster 2** | **Cluster 3** | **Cluster 4** | **Cluster 5** | **Cluster 6** | **Cluster 7** | **Cluster 8** |
| --- | --- | --- | --- | --- | --- | --- | --- | --- | --- |
| Gamification concept-to-user communication | Direct (%) | 94 | 100 | 100 | 95 | 92 | 100 | 100 | 100 |
|  | Mediated (%) | 6 | 0 | 0 | 5 | 8 | 0 | 0 | 0 |
| User identity | Virtual character (%) | 0 | 0 | 0 | 0 | 0 | 5 | 0 | 0 |
|  | Self-selected identity (%) | 100 | 100 | 100 | 100 | 100 | 95 | 100 | 100 |
| Rewards | Internal (%) | 76 | 12 | 31 | 36 | 100 | 33 | 0 | 83 |
|  | Internal and external (%) | 18 | 12 | 0 | 32 | 0 | 0 | 0 | 0 |
|  | No (%) | 6 | 76 | 69 | 32 | 0 | 67 | 100 | 17 |
| Competition | Direct (%) | 47 | 30 | 0 | 5 | 0 | 5 | 0 | 0 |
|  | Indirect (%) | 41 | 35 | 0 | 11 | 23 | 0 | 0 | 33 |
|  | No (%) | 12 | 35 | 100 | 84 | 77 | 95 | 100 | 67 |
| Target group | Patients (%) | 0 | 0 | 6 | 5 | 8 | 24 | 0 | 0 |
|  | Healthy individuals (%) | 100 | 100 | 94 | 95 | 92 | 76 | 0 | 0 |
|  | Health professionals (%) | 0 | 0 | 0 | 0 | 0 | 0 | 100 | 100 |
| Collaboration | Cooperative (%) | 70 | 0 | 0 | 11 | 24 | 24 | 0 | 0 |
|  | Supportive only (%) | 18 | 41 | 6 | 16 | 38 | 14 | 7 | 33 |
|  | No (%) | 12 | 59 | 94 | 73 | 38 | 62 | 93 | 67 |
| Goal setting | Self-set (%) | 35 | 100 | 60 | 0 | 92 | 71 | 7 | 0 |
|  | Externally set (%) | 65 | 0 | 40 | 100 | 8 | 29 | 93 | 100 |
| Narrative | Continuous (%) | 41 | 24 | 0 | 21 | 77 | 100 | 13 | 0 |
|  | Episodical (%) | 59 | 76 | 100 | 79 | 23 | 0 | 87 | 100 |
| Reinforcement | Positive (%) | 53 | 35 | 91 | 89 | 62 | 100 | 0 | 0 |
|  | Positive-negative (%) | 47 | 65 | 9 | 11 | 38 | 0 | 100 | 100 |
| Persuasive intent | Compliance change(%) | 41 | 0 | 77 | 47 | 8 | 33 | 47 | 17 |
|  | Behavior change(%) | 53 | 100 | 23 | 42 | 38 | 48 | 40 | 0 |
|  | Attitude change(%) | 6 | 0 | 0 | 11 | 54 | 19 | 13 | 83 |
| Level of integration | Independent(%) | 88 | 65 | 89 | 11 | 69 | 90 | 100 | 50 |
|  | Inherent(%) | 12 | 35 | 11 | 89 | 31 | 10 | 0 | 50 |
| User advancement | Presentation only(%) | 100 | 94 | 63 | 84 | 62 | 100 | 93 | 17 |
|  | Progressive(%) | 0 | 0 | 34 | 11 | 38 | 0 | 0 | 83 |
|  | No(%) | 0 | 6 | 3 | 5 | 0 | 0 | 7 | 0 |

| a. All characteristics of one dimension sum up to 100% as characteristics are mutually exclusive and collectively exhaustive [1].  b. The percentage of a characteristic for a cluster is the number of applicable items divided by the size of the cluster.  Darker colors represent higher percentages  ^0%^ 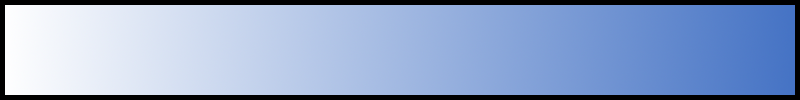 ^100%^ |
| --- |

## References

1. Nickerson RC, Varshney U, Muntermann J. A method for taxonomy development and its application in information systems. European Journal of Information Systems. 2013;22(3):336-59.
