# Supplementary material for: Archetypes of Gamification: Analysis of mHealth Apps
Source: JMIR Mhealth Uhealth. 2020 Oct 19;8(10):e19280. doi: 10.2196/19280 (PMC7605978; doi:10.2196/19280)
Supplement: Multimedia Appendix 9 [file mhealth_v8i10e19280_app9.docx]

## Multimedia Appendix 9. Relationship between archetype characteristics and targeted health behavior.

Table MA9-1. Number of mHealth apps targeting specific health behaviors per archetype.

|  | **A-1^a^** | **A-2^b^** | **A-3^c^** | **A-4^d^** | **A-5^e^** | **A-6^f^** | **A-7^g^** | **A-8^h^** |
| --- | --- | --- | --- | --- | --- | --- | --- | --- |
| # of **Physical Activity / Fitness** | 15 | 14 | 23 | 9 | 1 | 6 | 0 | 0 |
| # of **Overall Healthy Lifestyle** | 4 | 3 | 4 | 2 | 0 | 2 | 0 | 0 |
| # of **Nutrition** | 4 | 11 | 5 | 1 | 6 | 8 | 0 | 0 |
| # of **Female Health / Pregnancy** | 0 | 1 | 0 | 1 | 4 | 5 | 0 | 0 |
| # of **Mediation / Mental Health** | 0 | 2 | 5 | 2 | 5 | 3 | 0 | 0 |
| # of **Health Navigation** | 1 | 1 | 1 | 2 | 0 | 2 | 0 | 0 |
| # of **Therapy Adherence** | 0 | 4 | 2 | 2 | 2 | 6 | 0 | 0 |
| # of **Medical Education** | 2 | 1 | 8 | 3 | 0 | 0 | 16 | 5 |
| **Total** | 26 | 37 | 48 | 22 | 18 | 32 | 16 | 5 |
|  |  |  |  |  |  |  |  |  |

| Table MA9-1 shows the distributions of targeted health behaviors of mHealth apps per archetype. The 143 mHealth apps of the taxonomy are grouped according to their representativeness of one of the 8 archetypes. Each archetype is described by its two or three most significant characteristics. For a detailed listing of mHealth apps representing certain archetypes please refer to Multimedia Appendix 10.  a. Archetype 1: Physical Activity through Competition and Collaboration –  (competition = indirect, direct), (collaboration = supportive only, cooperative)  b. Archetype 2: Pursuing self-set Fitness goals without Rewards –  (rewards = no), (goal setting = self-set)  c. Archetype 3: Episodical Compliance Tracking –  (narrative = episodical), (persuasive intent = compliance change)  d. Archetype 4: Inherent Gamification for External Goals –  (goal setting = externally set), (level of integration = inherent)  e. Archetype 5: Self-set Goals for Mental Well-being –  (competition = no), (collaboration = supportive only, cooperative), (goal setting = self-set)  f. Archetype 6: Continuous Assistance through Positive Reinforcement –  (narrative = continuous), (reinforcement = positive)  g. Archetype 7: Medical Exam Preparation without Rewards –  (rewards = no), (target group health professionals)  h. Archetype 8: Learning through Progressive Gamification –  (target group = health professionals), (user advancement = progressive) |
| --- |

Figure MA9-1. Distribution of targeted health behaviors for Archetype 1.

Figure MA9-2. Distribution of targeted health behaviors for Archetype 2.

Figure MA9-3. Distribution of targeted health behaviors for Archetype 3.

Figure MA9-4. Distribution of targeted health behaviors for Archetype 4.

Figure MA9-5. Distribution of targeted health behaviors for Archetype 5.

Figure MA9-6. Distribution of targeted health behaviors for Archetype 6.

Figure MA9-7. Distribution of targeted health behaviors for Archetype 7.

Figure MA9-8. Distribution of targeted health behaviors for Archetype 8.
